# Genetic relatedness among *Neisseria gonorrhoeae* isolates in southeastern Michigan

**DOI:** 10.1017/ash.2023.314

**Published:** 2023-09-29

**Authors:** Anita Shallal, Robert Tibbets, Katherine Gurdziel, Dora Vager, Marcus Zervos, Geehan Suleyman

## Abstract

**Background:** Antimicrobial resistance (AMR) in *Neisseria gonorrhoeae* (NG) is an emerging public health crisis. Whole-genome sequencing (WGS) is an efficient way of predicting AMR determinants and their spread in the population. In a previous study, genotype–phenotype correlation analyses among NG isolates to determine antimicrobial resistance revealed discordance for azithromycin (AZM) and ceftriaxone (CRO) compared to other antibiotics. We investigated the evolutionary relatedness of NG isolated from patients with sexually transmitted infection (STI) using WGS in southeastern Michigan. **Methods:** Isolates, corresponding demographic data, and minimum inhibitory concentrations (MIC) via E-test (CRO) and broth microdilution (AZM) were obtained from the Michigan Department of Health and Human Services. Whole-genome libraries were prepared using the QIAseq FX kit followed by sequencing on a NovaSeq6000 (>200X Coverage); samples were aligned to NG reference strain TUM19854 (NZ_AP023069.1) using Snippy before phylogenetic tree generation using Neighbor-joining clustering on the core.aln files. Phylogenetic trees were visualized using ATGC:PRESTO. **Results:** In total, 38 isolates were analyzed. Demographic data and susceptibility testing results are noted in Table 1. Most isolates were from males (63%), Blacks (44.7%), individuals living in Detroit City proper (47.3%), and those with unknown HIV status (55.2%). More than one-third had prior STI, including NG. All isolates were susceptible to CRO (CLSI susceptible breakpoint MIC, 1). Within the phylogenetic tree, 8 main branches were identified (Fig. 1). Moreover, 1 branch contained a cluster with 12 closely related isolates, which included the 9 isolates with nonsusceptible AZM. Nearly all isolates in that cluster had been collected from Detroit City proper and Wayne County, suggesting epidemiological overlap and potential spread of resistant strains in those counties.

**Figure:**
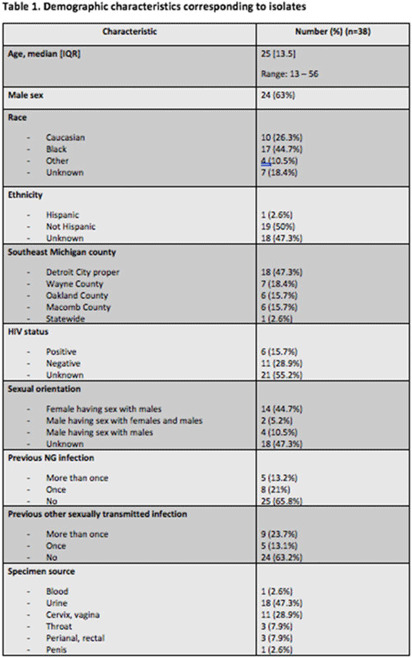

**Figure:**
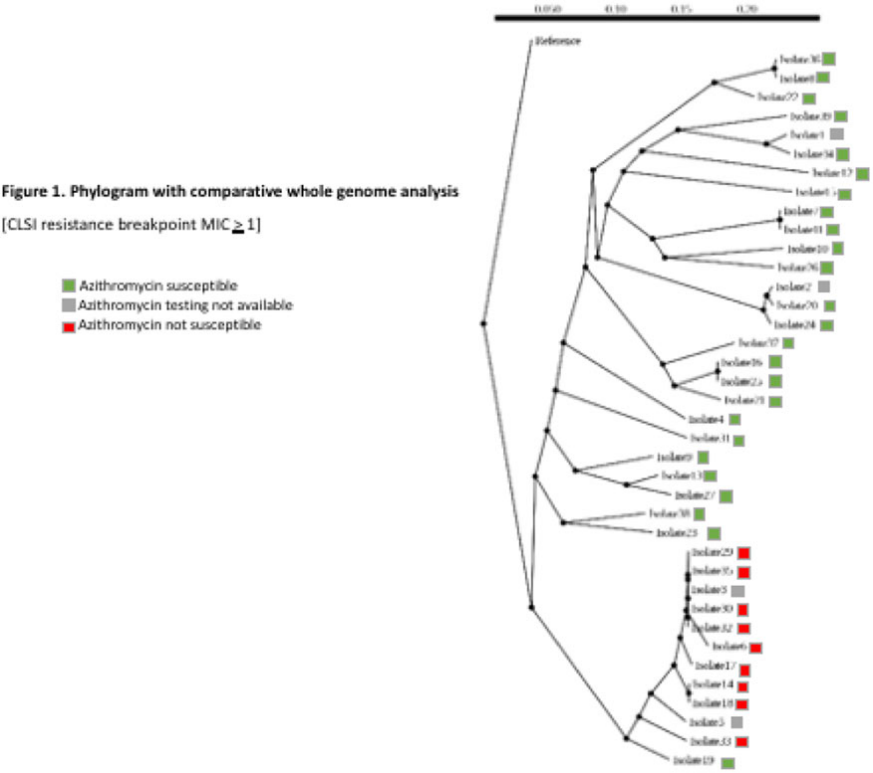

**Conclusions:** Comparative whole-genome and phylogenetic analyses among a subset of NG isolates revealed clustering of AZM resistance strains, suggesting a genomic component to AMR. Further studies are needed to determine the utility of WGS in diagnosis, outbreak investigations, and management of NG infections.

**Disclosures:** None

